# Profiling Chemicals Based on Chronic Toxicity Results from the U.S. EPA ToxRef Database

**DOI:** 10.1289/ehp.0800074

**Published:** 2008-10-20

**Authors:** Matthew T. Martin, Richard S. Judson, David M. Reif, Robert J. Kavlock, David J. Dix

**Affiliations:** National Center for Computational Toxicology, Office of Research and Development, U.S. Environmental Protection Agency, Research Triangle Park, North Carolina, USA

**Keywords:** cancer, chronic toxicity, pesticides, relational database, toxicity profile

## Abstract

**Background:**

Thirty years of pesticide registration toxicity data have been historically stored as hardcopy and scanned documents by the U.S. Environmental Protection Agency (EPA). A significant portion of these data have now been processed into standardized and structured toxicity data within the EPA’s Toxicity Reference Database (ToxRefDB), including chronic, cancer, developmental, and reproductive studies from laboratory animals. These data are now accessible and mineable within ToxRefDB and are serving as a primary source of validation for U.S. EPA’s ToxCast research program in predictive toxicology.

**Objectives:**

We profiled *in vivo* toxicities across 310 chemicals as a model application of ToxRefDB, meeting the need for detailed anchoring end points for development of ToxCast predictive signatures.

**Methods:**

Using query and structured data-mining approaches, we generated toxicity profiles from ToxRefDB based on long-term rodent bioassays. These chronic/cancer data were analyzed for suitability as anchoring end points based on incidence, target organ, severity, potency, and significance.

**Results:**

Under conditions of the bioassays, we observed pathologies for 273 of 310 chemicals, with greater preponderance (> 90%) occurring in the liver, kidney, thyroid, lung, testis, and spleen. We observed proliferative lesions for 225 chemicals, and 167 chemicals caused progression to cancer-related pathologies.

**Conclusions:**

Based on incidence, severity, and potency, we selected 26 primarily tissue-specific pathology end points to uniformly classify the 310 chemicals. The resulting toxicity profile classifications demonstrate the utility of structuring legacy toxicity information and facilitating the computation of these data within ToxRefDB for ToxCast and other applications.

The U.S. Environmental Protection Agency (EPA) and other regulatory agencies are investigating novel approaches to predict chemical toxicity, with the major goals being to enable the rapid screening of thousands of chemicals that have not previously been characterized, to increase mechanistic understanding of chemical toxicity, and to reduce the number of animals required for toxicity testing. All of these goals initially require high-quality *in vivo* toxicity data in order to test and validate these new approaches. To support U.S. EPA’s ToxCast effort (Dix et al. 2007), we have created the structured and curated Toxicity Reference Database (ToxRefDB) to tabulate information from guideline *in vivo* toxicity studies. ToxRefDB and related databases will help support computational analysis and modeling of the links from molecular interactions through cellular and organ phenotypes all the way to whole-animal toxicity. This transformation of existing toxicity data will facilitate a transition to the National Research Council’s (NRC) vision for *Toxicity Testing in the 21st Century* (Collins et al. 2008; NRC 2007). The NRC envisions a focus on toxicity pathways that will link molecular assays to toxicity outcomes in humans and ecological species.

Traditional toxicity testing for risk assessment of single compounds or limited groups of compounds can cost millions of dollars per chemical and years of effort. Since 1970, the U.S. EPA has accumulated a vast store of high-quality regulatory toxicity information on thousands of compounds, most of which has been inaccessible for computational analyses. The curation and structuring of chemical toxicity information into the readily accessible ToxRefDB have created a valuable resource for both retrospective and prospective toxicologic studies. ToxRefDB initially focused on capturing developmental rat and rabbit, multigeneration reproduction rat, and chronic/cancer rat and cancer mouse studies. In addition to the data model, we developed a detailed toxicity-based controlled vocabulary for all the study types spanning clinical chemistry, pathology, reproductive, and developmental effects.

An important initial application of ToxRefDB is to provide anchoring of *in vivo* toxicity data for the U.S. EPA’s ToxCast research program, which has been designed to address the agency’s needs for chemical prioritization by using state-of-the-art approaches in high-throughput screening (HTS) and toxicogenomics (U.S. EPA 2008b). Nearly all of the ToxCast phase I chemicals are food-use pesticide active ingredients that have undergone a full suite of mammalian toxicity tests, creating an unparalleled reference set of toxicologic information. The complete and highly standardized data set provided by ToxRefDB facilitates analysis of the ToxCast phase I chemicals across chemical, study type, species, target organ, and effect. Additionally, ToxRefDB serves as a model for other efforts to capture quantitative, tabular toxicology data from legacy and new studies and to make these data useful for cross-chemical computational toxicology analysis.

## Methods

### Data characteristics

We collected reviews of registrant-submitted toxicity studies, known as data evaluation records (DERs), for roughly 400 chemicals from the U.S. EPA’s Office of Pesticide Programs (OPP) within the Office of Pollution Prevention and Toxic Substances (OPPTS). The file types of the DERs include TIFF, Microsoft Word, Word Perfect, and PDF formats, some of which are not directly text-readable. We indexed every DER file based on a file name convention that consisted of the pesticide chemical (PC) code, study identification number (MRID), study type identification number [based on 870 series OPPTS harmonized health effect guidelines (U.S. EPA 1996)], species code, review identification number (TXR), and a review version code. The latter code identified the review as a primary review, secondary review, supplemental review, updated executive summary, or a deficient review.

For the initial build of ToxRefDB, we collected and indexed a total of 4,620 DERs from OPP. These included five types of studies from a variety of species: developmental in rat and rabbit, reproductive in rat, subchronic in mouse and rat, and chronic or cancer in rat and mouse. Approximately 1,000 DERs provided chronic and cancer data, and we selected a subset of these for curation into the database to yield data on 310 unique chemicals: rat chronic/cancer studies on 283 chemicals, and mouse cancer studies on 267 chemicals. Each study assessed a single technical-grade chemical’s toxicity potential in a single species and study type. The first portion of the DER outlines the test substance, purity, lot/batch numbers, MRID, study citation, OPPTS test guideline, and reviewers of the study. The executive summary captures all of the basic study design information, including species and strain, doses, number of animals per treatment group, and any deficiencies in study protocol.

Dose levels are listed in parts per million and through food consumption and body weight calculation or standard conversion as milligrams per kilogram body weight per day. Where possible, dose levels were listed as milligrams per kilogram body weight per day in ToxRefDB. The executive summary also describes adverse effects observed at all dose levels in the study. No observed adverse effect level (NOAEL) and lowest observed adverse effect level (LOAEL) are established based on adverse effects. The adverse effects used to derive NOAEL and LOAEL are referred to as “critical effects” in this article, regardless of their role in establishing reference dose levels in regulatory determinations for a chemical.

The body of the DERs provides detailed test material, animal information, and full dose–response data in text and tables for a variety of “effect types”, including mortality, clinical signs, clinical chemistry, hematology, urinalysis, gross pathology, nonneoplastic pathology, and neoplastic pathology. For each effect type, we also specified an “effect target” (e.g., liver as target organ) and “effect description” (e.g., hypertrophy).

ToxCast phase I chemicals also included nonpesticidal chemicals such as perfluorinated compounds, phthalates, and other industrial chemicals. Although DERs and pesticide registration studies were not available for these chemicals, there were often high-quality and standardized chronic and other types of toxicity studies available from the National Toxicology Program, peer-reviewed literature, or other sources. We organized and evaluated data from these study reports and publications consistent with the information from the DERs.

Information on chemical identity and structure was provided by the U.S. EPA DSSTox (Distributed Structure-Searchable Toxicity) program (U.S. EPA 2007). ToxRefDB outputs are linked to information from other sources through the U.S. EPA ACToR (Aggregated Computational Toxicology Resource) database (Judson et al. 2008b; U.S. EPA 2008a). ACToR will also serve as the primary portal for public access to ToxRefDB and related outputs. ACToR stores the HTS data being generated by the ToxCast program and will link these HTS data with traditional toxicity data from ToxRefDB and other sources.

### Relational model

In the development of ToxRefDB, a relational model approach was taken with input from other toxicity database standards, including ToxML (Yang et al. 2006). The resulting data model is semi-hierarchical in nature: a single compound can be tested in multiple studies, each study can contain multiple treatment groups, and multiple effects can be observed in each treatment group. The data model is organized from a chemical-centric viewpoint to allow data integration and exchange with other data sources and to facilitate the linkage of the reference toxicity information to chemical-specific data generated using *in vitro* technologies (i.e., ToxCast). The relational model was then implemented into a table structure with established relationships that ensure data integrity, updateability, and standardization [see Supplemental Material, Figure 1 (http://www.ehponline.org/members/2008/0800074/suppl.pdf].

### Development of a toxicity-based controlled vocabulary

The development of a controlled vocabulary within ToxRefDB was necessary for the standardization of data captured across various studies and study types performed over roughly 30 years. The nonredundant list of terms across various information domains provided data integrity and searchability. We based study type terminology on the unique study types harmonized by the Organisation for Economic Co-operation and Development and the OPPTS (U.S. EPA 1996). Specificstandardized terminology for study design was established for species/strain, method/route of administration, and units for dose and dosing duration. Treatment group-related vocabularies were developed to establish the generation, gender, and dosing period.

A primary goal in evaluating the registrant-submitted toxicity studies is to establish NOAEL and LOAEL values for a variety of categorical end points, including systemic, off-spring, maternal, parental, developmental, and reproductive toxicity across the various study types. These categorical end points are captured and normalized across studies for each effect responsible for deriving the NOAEL/LOAEL.

The development of a toxicologic effect vocabulary was approached in a domain-specific manner. For example, we derived clinical pathology terms from OPPTS guidelines and collected clinical pathology laboratories and organ pathology terms from various public resources, including the National Toxicology Program’s Pathology Code Tables (2007). The vocabulary underwent further standardization by mapping all synonymous terms to a single nonredundant value. We took a taxonomical approach for establishing the finalized effect vocabulary based on a three-tiered hierarchical model, with the effect type at the top, followed by effect target and then effect description. Examples of effect type include clinical chemistry, hematology, urinalysis, body weight, mortality, gross pathology, nonneoplastic pathology, neoplastic pathology, and developmental and reproductive effects. Subclasses of these types include specific target organs (e.g., liver, lung, spleen) or measured analytes (e.g., alanine aminotransferase, aspartate aminotransferase, cholesterol). The specific combinations of effect type and target are then further subclassed based on a nonredundant descriptive term (e.g., increase, decrease, hypertrophy, atrophy). For organ pathology terms, each target organ has a set of regions, zones, and cell types that characterize the site of toxicity. The full effect vocabulary is available on the ToxRefDB home page (U.S. EPA 2008c).

### Data input

The ToxRefDB Data Entry Tool was developed with Microsoft Access providing the user interface for all initial data input and is also available at the ToxRefDB home page (U.S. EPA 2008c). After the initial quality control (QC) steps discussed below, the data are migrated to ToxRefDB, which is implemented using the open-source MySQL platform. Data entry followed a series of protocols outlined in the ToxRefDB Standard Operating Procedure (SOP) documents that define mapping of toxicologic information to standardized fields, use of a standardized vocabulary, and extraction of biologically and statistically significant treatment-related effects.

### Data QC and management

QC consisted of 100% cross-checking of studies, systematic updates of ToxRefDB to ensure consistency across the studies, expert review of data outputs, and external review by stakeholders. All data entered into ToxRefDB have undergone cross-checking, which entailed a second person validating each entered value based on the source information (primarily DERs). Systematic QC involved querying the database for potential inconsistencies (e.g., male-only effects being assigned to female treatment groups, or systemic LOAEL being set at multiple dose levels) along with updating vocabularies and related records. Expert review was performed on data outputs of the chronic/cancer rat or mouse studies, including all of the end points captured in the data tables of this publication. In addition to internal QC, an ongoing process allowing stakeholders the opportunity to review ToxRefDB records is in place. The companies or registrants that sponsor the data or support the registration of the chemical are reviewing the accuracy of the data relative to DERs and other risk assessment documents. To date, studies on 235 chemicals have been reviewed by registrants, and comments from these reviews indicate greater than 99% accuracy in capturing treatment-related effects from DERs. The stakeholder review process has facilitated additional information from additional studies, DERs, and other risk assessment documents to be collected and entered into ToxRefDB.

### Data output and analysis

The structured toxicity information stored within ToxRefDB can be extracted in various formats using MySQL queries. For the purpose of providing computable outputs, that is, quantitative outputs amenable to statistical analysis, we used a consistent data output. The cross-tabulated data output consisted of rows of chemical information (e.g., CAS registry number and chemical name) and columns of end points or effects, with the cross section being the lowest dose at which the effect or end point was observed, that is, lowest effect level (LEL) in mg/kg/day. Even though NOAEL/LOAEL values can be queried from the database, the current analysis uses LELs, which do not reflect the NOAEL/LOAEL regulatory determinations derived from the studies and refer only to the minimum dose at which a specific effect or group of effects occurs. We used administered dose levels rather than molar concentrations to represent the chemically induced effects and end points, because of uncertainties in the pharmacokinetics linking administered dose to tissue concentrations reinforcing the fact that molecular weight alone cannot substitute for dosimetry. Additional transformation of the dosing information was performed, including log-based and binning methods for potency. For example, we developed a binning method for illustrating relative potency to provide information into the sensitivity of the end point from the perspective of treatment dose. To derive nonarbitrary dosing intervals, LEL for body weight changes were analyzed and separated into equivalent quintile bins (data not shown). The resulting bins, ≤ 15, ≤ 50, ≤ 150, ≤ 500, and > 500 mg/kg/day, were then applied to all end points. For instance, a chemical that caused liver hypertrophy at 5 mg/kg/day would be assigned a 5, at 25 mg/kg/day a 4, and so on. If the effect was not observed, then a zero was assigned. Additionally, log-transformed potency values were derived using –log_2_ of LEL. We used log_2_ to reflect the minimal dose spacing, that is, doubling, typically used for *in vivo* toxicology studies. A constant value of 12 was then added to zero-center the data, allowing for zero to represent no observed effect. Therefore, a value of 1 would be equivalent to an effect at 2,048 mg/kg/day and 18 would be equivalent to 0.015625 mg/kg/day. The resulting data formats are highly amenable to statistical data analysis, including descriptive and predictive data-mining algorithms.

We carried out unsupervised two-way hierarchical clustering across all chemicals of all effects with incidence greater than 5, as well as selected end points, based on log-transformed potency values using Pearson’s dissimilarity measure for both chemicals and effects. This analysis used Ward’s method for linkage (Ward 1963) and the agglomerative clustering method as implemented in the Partek Discovery Suite (Partek Inc., St. Louis, MO). In order to assess statistically significant species concordance across different effects, a permutation study was carried out. For each effect, the association between chemical and effect for the corresponding rat and mouse study was randomly permuted 1,000 times. We recorded the cross-species concordance for all simulations (permutations) and compared it with the observed concordance, thus giving an estimate of the concordance due purely to chance. Analyses were carried out using R version 2.6.1 (Ihaka and Gentleman 1996).

An initial 10% incidence cutoff was used to filter out individual and groups of effects for potential use in predictive modeling. This cutoff was chosen following the results of a related simulation study that demonstrated high levels of sensitivity and specificity for various machine learning methods on data with at least a 10% hit rate for predicted end points (Judson et al. 2008a). For other applications, it may be useful to add less frequently occurring effects and end points.

## Results

### Summary profiles of the ToxRefDB chronic/cancer data set

To date, ToxRefDB has captured *in vivo* mammalian toxicity study information from DERs for 411 conventional pesticide active ingredients. This present analysis focuses on the systemic toxicity and cancer end points culled from chronic/cancer rat or mouse studies on 310 of the chemicals entered into ToxRefDB. ToxRefDB enabled analysis to be performed along toxicologically related axes, including by chemical, study type, species, and effect. Study duration, dosing methods, data quality, guideline adherence, and sex were additional parameters for data filtering. In looking across all chronic/cancer rat and mouse studies, we assigned 19,537 effects to 3,082 different treatment groups in a total of 577 studies on 310 chemicals (Table 1). Effects are a combination of study type, species, effect type, effect target, and effect description for a given chemical, for example, chronic/cancer, rat, neoplastic pathology, liver, and adenoma. Across the 19,537 effects, 1,135 unique effects were observed, of which 484 were deemed critical effects, that is, criteria for establishing NOAEL/LOAEL, in at least a single study.

**Figure 1:**
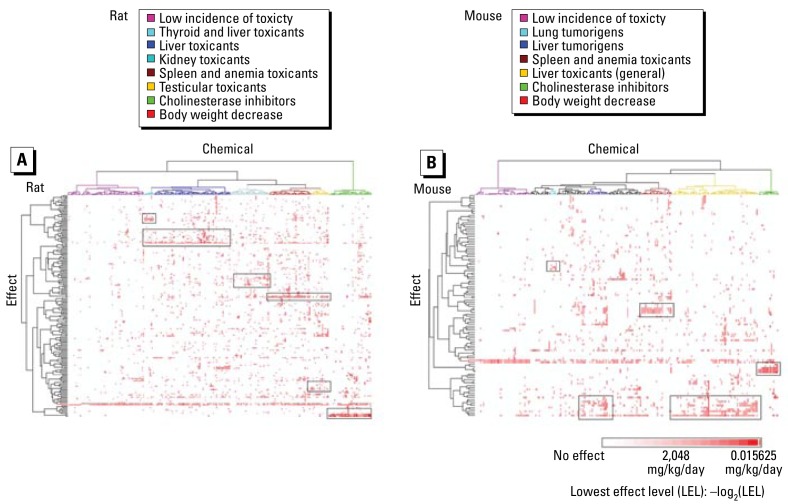
Unsupervised two-way hierarchical clustering of 207 effects in rat (*A*) and 112 effects in mouse (*B*) with incidence > 5, for 310 chemicals with chronic/cancer toxicity data in ToxRefDB. Specific clusters or classes based on associated toxicities are indicated by the color-coded chemical dendrogram: seven clusters for rat, and six for mouse.

The ToxRefDB chronic/cancer data set on 310 chemicals contained approximately 20,000 observed effects in rat or mouse studies. We achieved a high-level view of a subset of these data, and the relationships among chemical, effect, and potency, by unsupervised two-way hierarchical clustering of 207 rat (Figure 1A) and 112 mouse (Figure 1B) effects. For the rat, the 283 chemicals separated into seven distinct clusters or classes of the chemicals based on these toxicity profiles. Approximately 70 chemicals formed a cluster with an overall low incidence of toxicity, whereas the remaining chemicals displayed a unique set of toxicologic properties. More than 80 chemicals clustered as hepatotoxicants, and a subset of these also caused thyroid toxicity. Ten of the 15 conazole fungicides analyzed were in this hepatoxicity cluster. Clusters of chemicals exhibiting kidney, spleen/anemia, or testicular toxicities were not enriched for a specific chemical structural class. Cholinesterase inhibitors clustered separately from other chemicals and were enriched for organophosphates. In mouse, the 267 chemicals included clusters of cholinesterase inhibitors, spleen/anemia toxicants, and hepatotoxicants comparable with that observed for rat. Of the 112 total effects clustered in the mouse, 28 of these were liver toxicities, demonstrating the predominance of the liver as a target organ in the mouse. The unsupervised clustering of rat and mouse effects identified concentrations of effects and chemicals that were emphasized in subsequent, expert-driven approaches to chemical classification.

### Toxicity-based classification of chemicals

**Figure 2:**
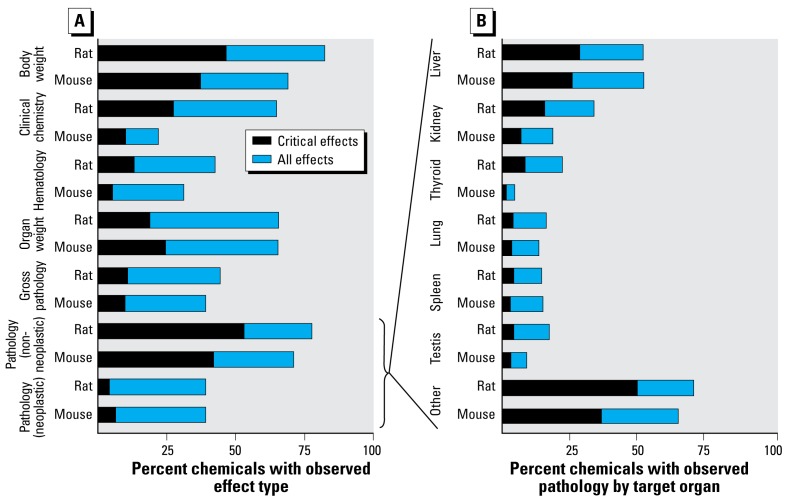
ToxRefDB chronic/cancer incidence data summarized by effect type (*A*) and by target organ pathology (*B*) for 310 chemicals with rat or mouse studies. Blue bars, total percentage of chemicals with that observed effect; black bars, percentage of chemicals for which that effect was used to derive systemic NOAEL/LOAEL levels.

The distribution of effects across effect types (Figure 2A) revealed that nonneoplastic pathologies dominate determination of systemic NOAEL/LOAEL, demonstrating the potential importance of this class of effects or end points to chemical regulation. The percentage of chemicals positive for an end point in both rat and mouse, over the total positive for the same end point in only the rat or mouse, was defined as “species concordance.” Species concordance for nonneoplastic pathology was 68%. Of the 167 chemicals that caused neoplastic lesions in rat or mouse chronic/cancer studies, 35% caused neoplastic lesions in both rat and mouse. We observed one or more pathologies in 273 of the 310 chemicals. The incidence of pathologic response, analyzed by target organ and species, was used to identify target organs for further investigation (Figure 2B). More than 90% of those 273 chemicals caused pathologies in the liver, kidney, thyroid, lung, testis, or spleen.

Whereas individual effects relating to highly detailed pathologic outcomes would provide classifications with the highest biological specificity, the limitations of classifying chemicals based solely on specific individual effects was apparent early in the analysis of ToxRefDB data. Only 11 specific, individual pathologic effects were observed for more than 10% of the chemicals (Table 2). Liver hypertrophy is the only common effect across both species based on a 10% incidence cutoff. In addition to low incidences of detailed pathologic effects, biases based on study design and pathology nomenclature limited the overall ability to compare chemical toxicities when we used individual effects. Grouping or aggregating related or near-synonymous terms, such as liver adenoma, combined adenoma/carcinoma, and carcinoma, resulted in more informative and statistically powerful sets of effects. Thus, the limitations of classifying chemicals based solely on specific individual effects were addressed by creating biologically related groupings of effects.

### Grouping tumor end points and extending to include proliferative lesions

This aggregative approach was illustrated by creating groups of neoplastic end points and the extension of these groups to include nonneoplastic proliferative lesions. The aggregation of neoplastic effects for each target organ resulted in an increase in the number of useful groupings beyond the individual mouse liver tumor effects shown in Table 2. However, the end points were still limited to mouse liver and rat thyroid neoplasia, based on an initial > 10% incidence cutoff. Associating the neoplastic end points with proliferative lesions increased the number of target organs to include liver, kidney, thyroid, lung, and testes. In general, only neoplastic lesions are considered indicative of rodent carcinogenicity. However, including nonneoplastic proliferative lesions provides a conservative model for assessing and predicting rodent tumorigenic potential, based on the assumption that prolonged proliferative response leads to eventual tumor formation. A simulation study was performed to assess whether the concordance between rat and mouse effects occurred at a rate greater than chance across neoplastic and proliferative classifications. Extending tumorigenicity groupings to include proliferative lesions significantly increased species concordance across numerous target organs, including the liver and kidney [see Supplemental Material, Figure 2 (http://www.ehponline.org/members/2008/0800074/suppl.pdf)].

### Mapping of toxicity end points to a cancer progression schema

**Figure 3:**
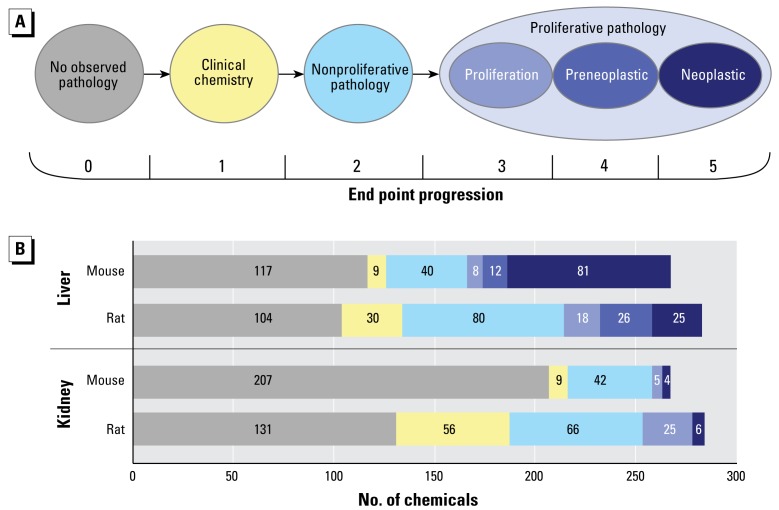
(*A*) ToxRefDB systemic toxicity and cancer outcomes represented along an end-point progression continuum. This schema was used to derive a severity score for each chemical based on the maximum value within a target organ. (*B*) Based on end-point progression, 310 chemicals were scored for liver and kidney pathology in rat and mouse chronic/cancer studies. Clinical chemistry used in this analysis is limited to target-organ–specific analytes (e.g., alanine aminotransferase for liver, and urea nitrogen for kidney).

Relationships between effects and the relative severity of those effects are not inherent to the database structure. Figure 3A presents a conceptualization of the end point progression schema in which chemicals were scored from 0 to 5 for each target organ, based on the severity of the effect, ranging from no observed pathology (0) to neoplastic lesions (5). End-point progression scoring reduced the possible chemical classifications to a single ordinal score (i.e., scores 0–5) for each target organ. Figure 3B presents the distribution of end-point progression scores for rat and mouse, liver and kidney. Examples of the impact of this scoring system include resmethrin, which caused treatment-related increases in a preneoplastic lesion (i.e., hyperplastic nodules) in the liver without progressing to a tumor. In contrast, metaldehyde caused treatment-related increases in liver tumors but was not identified as causing any preneoplastic lesions, even though preneoplastic lesions can be assumed to have occurred as a precursor event to liver tumor formation. Using the end-point progression scoring system allowed reasonable comparison of these two chemicals, if desired, by linking the preneoplastic score of 4 for resmethrin, to the neoplastic score of 5 for metaldehyde, along the continuum of end-point progression. The incidence of liver pathology between rats and mice was comparable when we grouped end-point progression scores. More than 50% of the chemicals tested resulted in a range of nonneoplastic to neoplastic lesions (i.e., scores 2–5). However, the relative severity for liver pathologic progression in mice was higher than in rats: 25 chemicals caused rat liver tumors, whereas 80 chemicals caused mouse liver tumors.

### Selected end points for predictive modeling

In addition to end points specific to various target organs, chemicals were classified with respect to multigender, multisite, or multispecies tumorigenicity (Table 3). Of the 310 chemicals in the chronic/cancer data set for which 240 chemicals were tested in both species, 167 chemicals were classified as tumorigens; 109 of those chemicals were multi gender, multisite, or multispecies tumorigens. Of the 283 chemicals tested in the rat, 42 chemicals were classified as multigender and multisite tumorigens. Of 267 chemicals tested in the mouse, 57 and 25 chemicals were classified as multigender and multisite tumorigens, respectively. Of the 240 chemicals tested in both species, 49 chemicals were classified as multispecies tumorigens. The distribution of relative potency values indicated that the rat was commonly more sensitive than the mouse for multigender and multisite tumorigenicity. In the rat, 38% of the multigender and 45% of the multisite incidences were at ≤ 50 mg/kg/day (i.e., relative potency values of 4–5), compared with 23% and 28% in the mouse. Conversely, 39% multigender and 28% multisite tumorigenicity occurred in the mouse at > 500 mg/kg/day (i.e., relative potency value 1), compared with 17% and 10% in the rat. Multispecies tumorigenicity was not achieved at doses ≤ 15 mg/kg/day, and 41% of incidences occurred at > 500 mg/kg/day.

**Figure 4:**
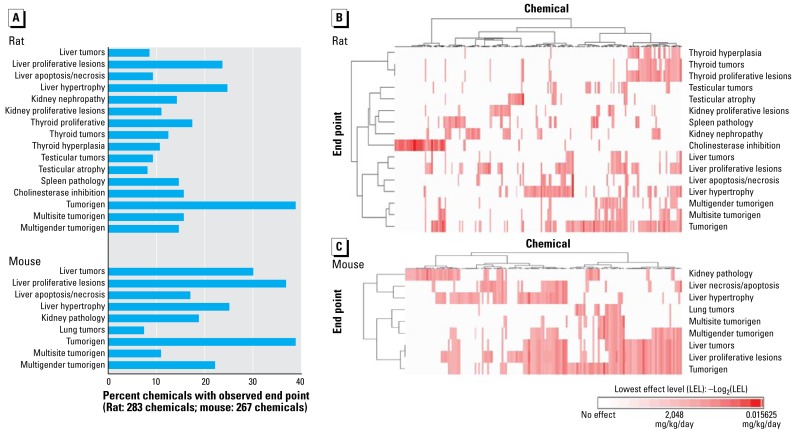
(*A*) The 16 rat and 9 mouse ToxRefDB end points from chronic/cancer studies selected for ToxCast predictive modeling. Two-way hierarchical clustering of the rat (*B*) and mouse (*C*) end points based on log-transformed potency values. Dose and potency values for all chemicals relative to these 25 end points are provided on the ToxRefDB home page (U.S. EPA 2008c).

Unsupervised and expert-driven approaches to end-point selection and subsequent chemical classification yielded near identical sets of target organs from which to select specific effects or aggregated effects. Based on incidence, severity, potency, and significance, 25 end points from chronic/cancer rat and mouse studies were selected for subsequent ToxCast predictive modeling (Figure 4A). The addition of multispecies tumorigens raised the total to 26 end points, each caused by 20 or more chemicals. Besides the multispecies tumorigen end point, 16 of the end points were from rat studies and 9 end points were from mouse. The same four end points were characterized in both rat and mouse liver, affording direct comparisons across species for tumors, proliferative lesions, apoptosis/necrosis, and hypertrophy. The only other frequent target organ common to both species was the kidney. Frequent rat-specific target organs included thyroid, testis, and spleen, whereas the only target organ specific to mouse was the lung. Unsupervised hierarchical clustering of the 16 rat end points (Figure 4B) and the 9 mouse end points (Figure 4C) displayed the relative distribution of the selected end points and chemicals. Of the 283 chemicals with a rat chronic/cancer study, 218 were positive in at least one of the selected end points, whereas 155 of 276 chemicals with a mouse cancer study were positive in at least one selected end point. Rat and mouse end points clustered primarily by target organ, with distinct clusters of thyroid, spleen, kidney, and liver toxicants in the rat. The high incidence of liver tumorigens in the mouse drives chemical groupings. However, chemicals causing or not causing liver hypertrophy and necrosis appear to segregate into two large groups of liver toxicants. In both species, the selected chronic/cancer end points represent the robust patterns of toxicologicresponse shown in Figure 2A and B. A full listing of the chronic/cancer end points derived from ToxRefDB for ToxCast predictive modeling, with their associated LELs, log-transformed potency, and relative potency values, are available on the ToxRefDB home page (U.S. EPA 2008c).

## Discussion

Advancing alternative testing methods for assessing chemical safety requires an informed transition from the current toxicity testing to systems that are higher throughput, more predictive, and not as dependent on the extensive use of animals. To support this transition, we created ToxRefDB to capture a rich set of existing *in vivo* laboratory animal toxicity data on a group of environmentally relevant, well-studied chemicals. Pesticide active ingredients have comprehensive toxicity profiles that are opportune data sets for creating a bridge from *in vivo* to *in vitro* toxicology. ToxRefDB digitizes and stores toxicity data in a structured and searchable format, and using structured data mining methods makes these data a computable resource for predictive toxicology efforts such as the U.S. EPA’s ToxCast program (U.S. EPA 2008b).

Individual toxicity effects based on unique type, target, and description yielded only a small number of *in vivo* end points across a significant number of chemicals supportive of robust predictive modeling. However, grouping effects by effect type and target often collapsed hundreds of individual effects into a single end point, common to dozens of chemicals. The goal was to strike a balance between maintaining biological specificity across a group of related effects while increasing total incidence for effects across a critical mass of chemicals. For example, extending tumor end points to include proliferative lesions increased not only total incidence but also species concordance and thus increased confidence in characterizing a chemical’s potential toxicity. Grouping proliferative lesions also addressed other potential factors, such as changes in pathology nomenclature over time (Wolf and Mann 2005) and reporting inconsistencies. Deriving end points based on groups of effects yielded organ- and species-specific end points in the liver, kidney, thyroid, testis, spleen, and lung in rats or mice with a high enough incidence across ToxRefDB chemicals to support predictive modeling.

Another approach for addressing the limitations of profiling chemicals based on individual toxicity effects was to compare the severity of these effects across a continuum of pathophysiology. Because the progression to cancer (Hanahan and Weinberg 2000) and organ-specific progression to tumorigenicity (Cohen and Arnold 2008) have been well characterized, we created a five-point severity scoring system to encode this. Using this approach, ToxRefDB provides a quantitative value associated with the key events in the progression to tumor formation and cancer. Incorporating additional information on the severity of *in vivo* effects in ToxRefDB may be fruitful in future modeling and predictive toxicology efforts. Additional data not currently in ToxRefDB, including incidence data, would have to be added for more detailed dose–response analyses and assessment of the magnitude of change for specific effects.

Because many of the tumors caused by chemical exposure in ToxRefDB occur at high doses that are many orders of magnitude removed from potential human exposures, it is useful to also consider multigender, multisite, and multispecies tumorigenicity in the course of evaluating chemicals. Current U.S. EPA cancer risk assessments use multisite and multispecies tumorigenicity as indicators of increased significance for tumor findings (U.S. EPA 2005). Thus, the tumorigenic end points selected for ToxCast predictive modeling included multigender, multisite, and multispecies tumorigens. Additional analyses of these multiplicities in the tumorigenicity data of ToxRefDB are under way, with the goal of improving hazard assessments, chronic/cancer study protocols, and future data requirements.

Success in predicting target-organ–specific effects in ToxCast will depend on numerous factors, including the target, species, and dose response of the effects that are being predicted. In the present analysis of ToxRefDB, we identified effects in the liver, kidney, thyroid, testis, spleen, and lung in rats or mice that we will now attempt to predict using *in vitro* data from ToxCast. Because species concordance of the *in vivo* effects in ToxRefDB was fairly limited, success in predicting species-specific versus multispecies effects will be an interesting outcome of ToxCast. The dose responses for selected end points are also provided by ToxRefDB, including log-transformed potency values conducive to computational analysis, and relative potency values that facilitate comparisons across chemicals and end points. These quantitative data should facilitate development of new *in vitro* and *in silico* methods to predict *in vivo* chemical toxicity.

Although numerous studies have evaluated the use of biochemical, cell-based, and genomic assays to build predictive models of toxicity, these efforts have usually been limited to only a partial view of the complex biology underlying tissue, organ, or whole-animal toxicity. By probing such a broad spectrum of biology in the hundreds of ToxCast assays, the “toxicity signatures” will be optimally predictive and representative of a broad range of *in vivo* toxicity end points. A variety of statistical techniques and machine learning approaches will be used to mine this complex data set for toxicity signatures with high sensitivity and specificity. These include linear discriminant analysis, support vector machines, and neural networks. In addition to these automated approaches, more hypothesis-driven, biologically based signatures will assist in filling the large gap between molecular and phenotypic end points. It is expected that assays of multiple types, probing multiple pathways, will be required to predict *in vivo* toxicity across a wide range of chemicals—this is the approach taken within ToxCast and ToxRefDB.

ToxRefDB continues to develop, adding toxicity end points from additional study types, including multigeneration reproductive and prenatal developmental tests, for predictive modeling in the ToxCast research program. Besides expanding toxicity coverage to other study types, ToxRefDB will expand in chemical coverage to include more nonpesticide chemicals. As each of these ToxRefDB data sets pass through U.S. EPA quality and clearance processes, they will be made publicly available through peer-reviewed publications, ToxRefDB home page, and ACToR. The contents of the entire database will be viewable and searchable in the future through a Web-based query tool located on the ToxRefDB website (U.S. EPA 2008c).

ToxRefDB offers unparalleled amounts of legacy toxicity information on environmental chemicals captured in a structured format, providing a platform for repeated and updated chemical characterizations. Creating the ability to search and filter across 30 years’ worth of toxicity data required extensive amounts of data normalization, annotation, and curation and was made possible through the development of a robust standardized vocabulary for the fields and data elements within ToxRefDB. In the present study, we used chronic toxicity data in ToxRefDB to derive toxicity profiles for the ToxCast phase I chemicals, yielding a set of toxicity-based and predictable end points. In future applications of ToxRefDB, researchers, risk assessors, and regulators will use the database for retrospective and modeling projects looking across a large landscape of chemical and toxicity space.

## Figures and Tables

**Table 1:** Summary statistics for chronic/cancer rat and mouse studies entered into ToxRefDB.

Study	Chemicals	No. of studies	Treatment groups	Treatment groups with effects	Effects^a^	Critical effects^b^
Total chronic/cancer	310	577	7,340	3,082	19,537	3,119
Rat	283	298	4,228	1,721	12,215	1,816
Mouse	267	279	3,059	1,344	7,416	1,303

a Total number of effect type, target, and description combinations assigned to any treatment group.

b Effects that are criteria for establishing the study-specific NOAEL/LOAEL.

**Table 2:** Pathology observed for > 10% of ToxRefDB chemicals in chronic/cancer rat and mouse studies.

Target organ	Effect	Percent observed
Rat		
Liver	Hypertrophy	25
Kidney	Nephropathy	14
Liver	Vacuolization	12
Thyroid	Adenoma	11
Thyroid	Hyperplasia	11
Mouse		
Liver	Hypertrophy	25
Liver	Adenoma	21
Liver	Necrosis	16
Liver	Adenoma/carcinoma combined	14
Liver	Pigmentation	14
Liver	Carcinoma	12

**Table 3:** Multigender, multisite, and multispecies tumorigens in ToxRefDB.

	Rat		Mouse				Rat		Mouse		
Chemical	Multigender	Multisite	Multigender	Multisite	Multispecies	Chemical	Multigender	Multisite	Multigender	Multisite	Multispecies
Carbaryl	2	2	1	1	2	Tribufos			3	4	
Dipropyl isocinchomeronate	1	1	1	1	1	Amitraz			3	3	
						Fenoxycarb			3	3	
Fentin	5	5	4		4	Spiroxamine			3	3	
Dazomet	5	5	3		4	Tefluthrin			3	3	
Clodinafop-propargyl	4	4	4		4	Permethrin			2	2	
Lactofen	3	3	5		4	Trifloxystrobin			2	2	
Dimethoate		5	4	4	4	Chloridazon			1	1	
Malathion	4	4	1		1	Triforine			1	1	
Diuron	4	3		1	1	Dichlorvos	5	5	N	N	N
Dacthal	2	2	1		1	Pyraclostrobin	5	5	N	N	N
Isoxaflutole	2	2	1		1	Alachlor	4	3	N	N	N
Spirodiclofen	2	2	1		1	Captan	N	N	3	3	N
Diclofop-methyl	4	4			4	Maneb	N	N	2	2	N
Cinmethylin		3	3		3	Azafenidin					4
Imazalil		3	3		3	Lindane					4
Nitrapyrin			3	3	3	Fluazinam					3
Propoxur	2	2			2	Paclobutrazol					3
Daminozide			2	1	2	Acephate					2
Thiacloprid	4	4			1	Linuron					2
Vinclozolin	3	4			1	Propanil					2
Di(2-ethylhexyl)phthalate	1		1		1	Triasulfuron					1
Folpet		5	1		1	Fipronil	4				
MGK (octacide 264)		2	1		1	Thiabendazole	3				
Iprodione			1	1	1	Boscalid	2				
Cacodylic acid	5				3	Pendimethalin	2				
Propyzamide		4			3	Pyrimethanil	2				
Oxadiazon			5		3	5,5-Dimethylhydantoin	1				
Resmethrin	2				2	Cyazofamid	1				
Pyrithiobac-sodium	1				2	Chloropicrin		5			
Bentazone		2			2	Fenamiphos		5			
Fluthiacet-methyl			5		2	Molinate		5			
Metaldehyde			2		2	Chlorpyrifos-methyl		4			
Triflusulfuron-methyl			2		2	Fluoxastrobin		1			
Fludioxonil				2	2	Fenitrothion			5		
Prodiamine	1				1	Cyproconazole			4		
Tepraloxydim		2			1	Prochloraz			4		
Clofencet-potassium		1			1	Thiamethoxam			3		
Isoxaben			1		1	Bispyribac-sodium			2		
Pymetrozine			1		1	Piperonyl butoxide			2		
Topramezone			1		1	Propiconazole			2		
Triadimefon			1		1	Acifluorfen-sodium			1		
Oryzalin	4	4				Difenoconazole			1		
Simazine	4	4				Primisulfuron-methyl			1		
Tebufenpyrad	4	4				Pyraflufen-ethyl			1		
Dichloran	3	3				Thiodicarb			1		
Dimethenamid	3	3				Fenoxaprop-ethyl				4	
Prosulfuron	3	3				Buprofezin				2	
Acetochlor	3	2				Propargite	4		N	N	N
Ametryn	2	3				Dichlobenil	2		N	N	N
Oxytetracycline HCl	1	1				Quintozene	2		N	N	N
Bifenthrin			5	5		Tralkoxydim		3	N	N	N
Disulfoton			5	5		Benomyl	N	N	3		N
Metam-sodium			4	5		Cloprop	N	N	2		N
Quizalofop-ethyl			4	4		Thiophanate-methyl	N	N	1		N

Relative potency: 5, ≤ 15 mg/kg/day; 4, ≤ 50 mg/kg/day; 3, ≤ 150 mg/kg/day; 2, ≤ 500 mg/kg/day; 1, > 500 mg/kg/day; N, not assessed (no study available).

## References

[b1-ehp-117-392] Cohen SM, Arnold LL (2008). Cell proliferation and carcinogenesis. J Toxicol Pathol.

[b2-ehp-117-392] Collins FS, Gray GM, Bucher JR (2008). Transforming environmental health protection. Science.

[b3-ehp-117-392] Dix DJ, Houck KA, Martin MT, Richard AM, Setzer RW, Kavlock RJ (2007). The ToxCast program for prioritizing toxicity testing of environmental chemicals. Toxicol Sci.

[b4-ehp-117-392] Hanahan D, Weinberg RA (2000). The hallmarks of cancer. Cell.

[b5-ehp-117-392] Ihaka R, Gentleman R (1996). R: a language for data analysis and graphics. J Comput Graph Stat.

[b6-ehp-117-392] Judson RS, Elloumi F, Setzer RW, Li Z, Shah I (2008a). A comparison of machine learning algorithms for chemical toxicity classification using a simulated multi-scale data model. BMC Bioinformatics.

[b7-ehp-117-392] Judson RS, Richard AM, Dix DJ, Houck K, Elloumi F, Martin MT (2008b). ACToR—Aggregated Computational Toxicology Resource. Toxicol Appl Pharmacol.

[b8-ehp-117-392] National Research Council (2007). Toxicity Testing in the 21st Century: A Vision and a Strategy.

[b9-ehp-117-392] National Toxicology Program (2007). Pathology Code Tables.

[b10-ehp-117-392] U.S EPA (U.S. Environmental Protection Agency) (1996). OPPTS Harmonized Test Guidelines.

[b11-ehp-117-392] U.S. EPA (2005). Guidelines for Carcinogen Risk Assessment Risk Assessment Forum.

[b12-ehp-117-392] U.S EPA (U.S. Environmental Protection Agency) (2007). Distributed Structure-Searchable Toxicity Database.

[b13-ehp-117-392] U.S EPA (U.S. Environmental Protection Agency) (2008a). ACToR Home.

[b14-ehp-117-392] U.S EPA (U.S. Environmental Protection Agency) (2008b). ToxCast Program.

[b15-ehp-117-392] U.S EPA (U.S. Environmental Protection Agency) (2008c). ToxRefDB Home.

[b16-ehp-117-392] Ward JH (1963). Hierarchical grouping to optimize an objective function. J Am Stat Assoc.

[b17-ehp-117-392] Wolf DC, Mann PC (2005). Confounders in interpreting pathology for safety and risk assessment. Toxicol Appl Pharmacol.

[b18-ehp-117-392] Yang C, Benz RD, Cheeseman MA (2006). Landscape of current toxicity databases and database standards. Curr Opin Drug Discov Dev.

